# Quality control processes in allografting: A twenty-year retrospective review of a hospital-based bone bank in Taiwan

**DOI:** 10.1371/journal.pone.0184809

**Published:** 2017-10-19

**Authors:** Shau-Huai Fu, Jyh-You Liu, Chuan-Ching Huang, Feng-ling Lin, Rong-Sen Yang, Chun-han Hou

**Affiliations:** 1 Department of Orthopedics, National Taiwan University Hospital Yun-Lin Branch, Yun-Lin County, Taiwan; 2 Department of Laboratory Medicine, National Taiwan University Hospital, Taipei, Taiwan; 3 Department of Dermatology, Cathay General Hospital, Taipei, Taiwan; 4 Department of Orthopedics, National Taiwan University Hospital, Taipei, Taiwan; University of Toledo, UNITED STATES

## Abstract

Musculoskeletal allografts are now commonly used. To decrease the potential risks of transmission of pathogenic bacteria, fungi, or viruses to the transplant recipients, certain issues regarding the management of patients who receive contaminated allografts need to be addressed. We aimed to clarify the incidence and extent of disease transmission from allografts by analyzing the allografting procedures performed in the bone bank of our hospital over the past 20 years. We retrospectively reviewed the data from our allograft registry center on 3979 allografts that were implanted in 3193 recipients throughout a period of two decades, from July 1991 to June 2011. The source of the allografts, results of all screening tests, dates of harvesting and implantation, and recipients of all allografts were checked. With the help of the Center for Infection Control of our hospital, a strict prospective, hospital-wide, on-site surveillance was conducted, and every patient with healthcare-associated infection was identified. Fisher’s exact test was used to compare the infection rate between recipients with sterile allografts and those with contaminated allografts. The overall discard and infection rates were, respectively, 23% and 1.3% in the first decade (1991–2001); and 18.4% and 1.25% in the second decade (2001–2011). The infection rate of contaminated allograft recipients was significantly higher than that of sterile allograft recipients (10% vs. 1.15%, *P* < 0.01) in the second decade. Both infection and discard rates of our bone bank are comparable with those of international bone banks. Strict allograft processing and adequate prophylactic use of antibiotics are critical to prevent infection and disease transmission in such cases.

## Introduction

Musculoskeletal allografts currently represent common practice in orthopedic surgeries including revision arthroplasty, spine surgery, and revision surgery for non-union. Following a rapid increase over the previous decade, approximately 1,300,000 grafts were used in the United States in 2003 [1], and the demand for musculoskeletal allografts has been growing.

However, disease transmission through grafts remains a serious problem. Several studies, including our previous study, have revealed that bacterial or viral infections originating from the allograft cause severe morbidity and mortality in the recipients [2–7]. Additional issues such as management of patients who received contaminated or infected allografts remain to be addressed.

Bone banking aims to provide safe and adequate allografts for reconstructive surgery. Previous reports regarding the performance of bone banks either did not distinguish between allografts from living and cadaveric donors [2,4,5,7], or presented cases with short-term follow-up [3]. Our bone bank is one of the largest single hospital-based bone banks in the world, and maintains the largest living donor series. Thus, we believe our allograft experience of over 20 years may offer valuable information on the state-of-the-art in bone banking. In order to gain more insight regarding the prevalence of disease transmission from allografts, we analyzed the performance of our hospital-based bone bank in terms of incidence, type, and severity of contamination.

## Materials and methods

In 1989, we established a hospital-based bone bank at the Orthopaedic Department of National Taiwan University Hospital; we followed the guidelines of the Centers for Disease Control and Prevention, USA. The bone bank has been working well since 1991, when a full-time employee was assigned to work there. In 2005, we published a 10-year review of our bone bank, describing the performance of the bone bank between July 1991 and June 2001 [6]. In the current study, we retrospectively reviewed data from the following decade (i.e., between July 2001 and June 2011), pertaining to 2614 allografts and 1840 recipients, and compared the results with those of our previous study [6]. We recorded the source of the allografts, the results of all screening tests, the dates of harvesting and implantation, and information about the recipient of each allograft. This study was approved by the Research Ethics Committee C of National Taiwan University Hospital (approval no. 201502017RINB), which waived the need for obtaining informed consent, as no patient identification data was included in the analysis.

We followed the well-established methods described in our previous report for harvesting, packing, and storing the allografts [6]. The laboratory screening examinations included hepatitis B virus surface antigen (HBsAg) test, venereal disease research laboratory (VDRL) test, anti-hepatitis C virus antibody (anti-HCV) test, anti-human immunodeficiency virus (anti-HIV) antibody test, and bacterial culture (first culture) from swab sample obtained when harvesting the allograft. The swab culture stick using BD BBL™ CultureSwab EZ™ (Sparks, MD, USA) was preserved in a dry sterile tubular container, thus preserving an adequate environment for transportation. The swab was transported to the culture laboratory within 30 minutes under ambient temperature. The culture was then plated on BD Trypticase™ Soy Agar with 5% Sheep Blood (BAP agar) for aerobic micro-organisms, and Dr. Plate Anaerobic Blood Agar with CDC formulation (CDC agar) and BD BBL™ CDC Anaerobe 5% Sheep Blood Agar with Phenylethyl Alcohol (CDC-PEA agar) for anaerobic micro-organisms, and subsequently bathed in a semisolid broth tube, GAM-semisolid, that enabled aerobic micro-organisms to grow in the upper layer and anaerobic in the lower layer. The aerobic agar was incubated at 35°C for three days in a 5% CO2 environment, and we checked for possible growth of micro-organisms at 24 and 72 hours. The anaerobic agar was incubated at 35°C for nine days in the glove box with anaerobic environment, and we checked for possible growth of micro-organisms at 48 hours and on the ninth day. The broth was incubated at 35°C for nine days in 5% CO2 environment, and we checked for possible growth of micro-organisms every day. In the second decade, a few changes were introduced. Our primary care residents screened the patient’s social and medical history, performed thorough physical examination, and obtained informed consent before surgery. These changes helped avoid allografts from unsuitable donors (S1 Appendix).

We did not use radiation or chemical sterilization to preserve osteoinduction and the biomechanical benefits of the allografts. All allografts were stored frozen at -70°C before implantation, and were used within 6 months of procurement. Another swab culture of the allograft was performed after thawing, during the implantation surgery. The swab culture was performed by surgical assistants on an isolated, sterile table in the operation theatre, where the allograft was processed to prevent further contamination. The swab culture methods were the same as those used during harvesting. Before implantation, all allografts were washed with a copious amount of sterile, warm, normal saline, and bathed in gentamicin solution (80 mg gentamicin in 200–300 mL of normal saline, with the concentration between 0.27–0.4 mg/ml). The recipients underwent preoperative skin preparation, and received an intravenous injection of prophylactic first-generation cephalosporin within 30 min of the operation, as ruled by the Joint Commission International.

With the help of the Center for Infection Control of our hospital, strict, prospective, hospital-wide, on-site surveillance was conducted [8]. Every patient with healthcare-associated infections was identified and registered in their database. We tracked every hospitalized patient who had developed infection after an allogenic bone graft transplantation by connecting the infection database and our bone bank database. Wound culture results were also obtained according to the medical records.

We used the Chi-square test to compare the discard rate and the data regarding HBsAg-positive donors in the first and second decades. The comparison of infection rates between recipients with sterile allografts and those with contaminated allografts was performed using Fisher’s exact test.

## Results

**Fig 1:**
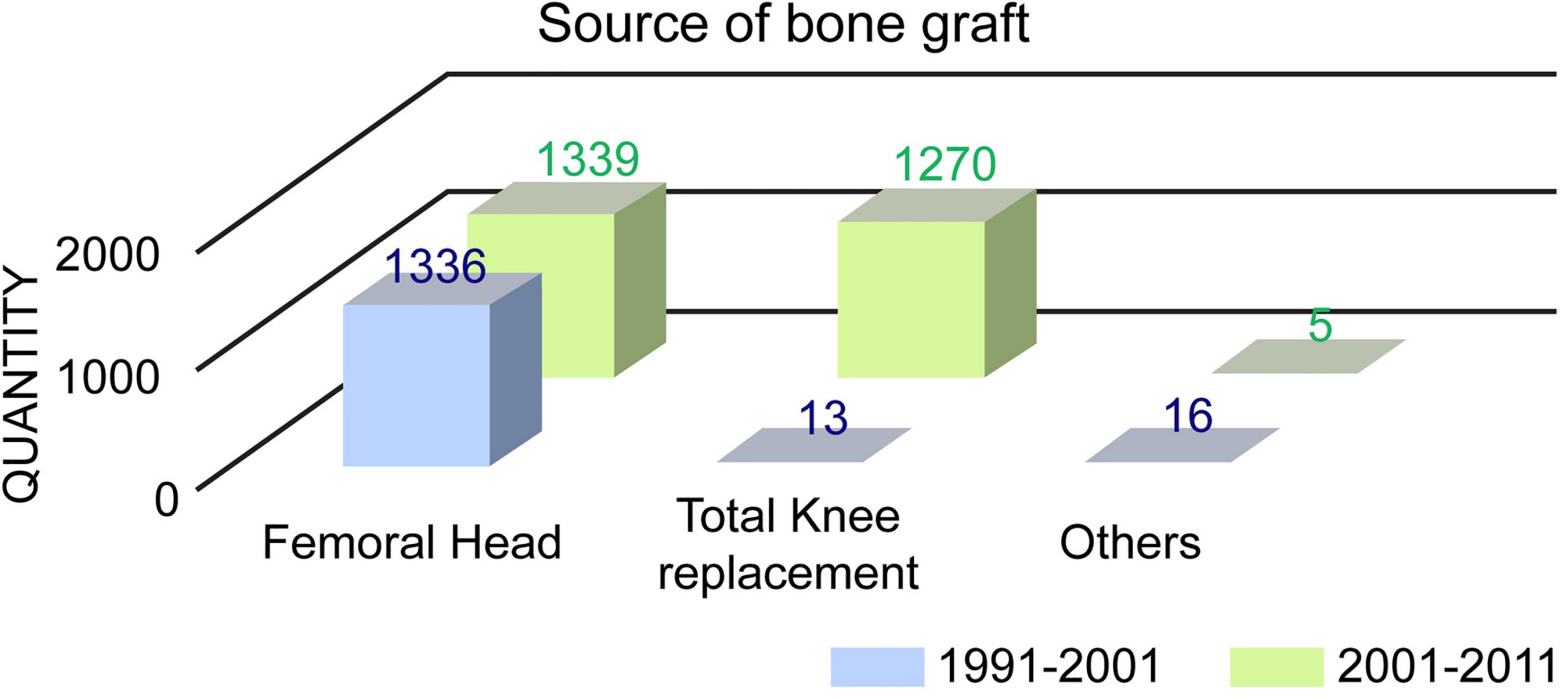
Source of bone graft. As the demand for allografts grew rapidly, more bone grafts were harvested during total knee replacement. Other sites for harvesting include the femoral and tibial condyle and proximal humerus.

During the first decade of activity of our bone bank (July 1991-June 2001), 98% of the allografts were obtained from the femoral head, which was harvested during total or hemi-hip arthroplasty [6]. As the demand for allografts increased rapidly, we began to use resected bones from total knee arthroplasty (TKA) as another important source of bone grafts. Consequently, in the second decade of activity of our bone bank (July 2001-June 2011), the number of allografts obtained from TKA (48.6%) almost equaled that of allografts obtained from hip arthroplasty (51.2%) (Fig 1).

Between July 2001 and June 2011, 3203 allografts were harvested. Of those, 589 were discarded for not passing the laboratory screening tests. The discard rate was 18.4% (589/3203), which is significantly lower than that noted for the previous decade, between July 1991 and June 2001 (23%; *P* = 0.01). The leading causes for discarding the allografts were that the donor serum was HBsAg positive (41.6%), anti-HCV positive (31.6%), positive for venereal disease (16.1%), bacterial culture positive (8.7%, described below), or anti-HIV antibody positive (2%). The decrease in the seroprevalence of HBsAg from 12.7% (212/1674 in the first decade, from July 1991 to June 2001) to 7.6% (245/3203 in the second decade, from July 2001 to June 2011) was statistically significant (*P* < 0.05). No significant differences were observed with respect to the results from the VDRL, as the discard rates were 2.9% (49/1674) and 3.0% (95/3203) for the first and second decade, respectively.

The microbiological contamination rate (derived from the first culture) of the allografts procured in the operating theatre was 1.6% (51/3203 in the second decade, from July 2001 to June 2011). In that group, coagulase-negative staphylococci (CoNS) were the most common pathogens (52.9%), followed by *Corynebacterium* sp. (9.8%), *Propionibacterium* sp. (5.9%), and *Staphylococcus aureus* (5.9%, 2 methicillin-sensitive *S*. *aureus*, and 1 methicillin-resistant *S*. *aureus*). The 2614 remaining allografts were implanted, of which 20 (0.77%) were positive for bacterial cultures after thawing. The most common pathogens were CoNS (40%), followed by *Bacillus* sp. (15%), *Propionibacterium acnes* (10%), and Gram-positive rods (10%).

According to the bone bank registry data, the 2614 allografts implanted in the second decade were used in 1840 recipients, of whom 1160 (63.0%) underwent spine surgery, 344 (18.7%) underwent revision total hip arthroplasty, 255 (13.9%) underwent revision surgery for non-union, and 81 (4.4%) underwent other procedures such as tumor surgery or arthrodesis. Among these 1840 allograft recipients, 23 cases (Table 1) of proven infection that need hospitalization with intravenous antibiotics treatment or surgical debridement were found, and the overall infection rate was 1.25%, which was comparable with that noted in our previous report (1.3%) [6]. The infection rate for revision total hip replacement was 2.03% (7/344).

**Table 1:** Pathogens identified in the allograft transplant cases with proven infection (n = 23).

Case N°.	Sex	Age (years)	Surgery	Wound culture	Swab culture after thawing	
1	F	88	Revision THR	MRSA		Negative
2	M	68	Revision THR	MSSA		Negative
3	M	64	Revision THR	MRSA		Negative
4	M	50	Tibia open fracture	MSSA		Negative
5	F	70	PDPIPF	*Enterococcus* spp.		Negative
6	F	52	Revision ORIF	MRSA, CoNS		Negative
7	F	12	PIPF for scoliosis	MRSA		Negative
8	F	55	Revision THR	CoNS		Negative
9	F	74	PDPIPF	CoNS		Negative
10	M	86	PDPIPF	*Escherichia coli*		Negative
11	M	41	PDPIPF	MRSA		Negative
12	F	82	Revision THR	*Escherichia coli*		*Enterococcus* spp.
13	F	68	PDPIPF	MRSA		CoNS
14	M	57	PDPIPF	CoNS		Negative
15	M	50	Revision ORIF	*Enterobacter cloacae*		Negative
16	M	56	Revision THR	MRSA		Negative
17	M	67	Revision ORIF	MRSA		Negative
18	M	51	Revision THR	Group B streptococcus		Negative
19	M	71	Tibia plateau fracture	MSSA, CoNS		Negative
20	F	67	PDPIPF	MRSA		Negative
21	M	67	Revision THR	*Acinetobacter baumannii*		Negative
22	F	60	Revision ORIF	*Enterobacter cloacae*		Negative
23	M	24	Tibia open fracture	*Klebsiella pneumoniae*^*a*^		Negative

^a^Extended-spectrum β-lactamase producing *Klebsiella pneumoniae*

THR: total hip replacement; PDPIPF: posterior decompression, posterior instrumentation, and posterior fusion; ORIF: open reduction and internal fixation; MRSA: methicillin-resistant *Staphylococcus aureus;* MSSA: Methicillin-sensitive *Staphylococcus aureus;* CoNS: coagulase-negative staphylococci

Detailed information regarding the allograft bone recipients who received the allografts that were positive for bacterial culture after thawing is summarized in Table 2. Twenty recipients received allografts that were positive for bacterial culture after thawing; two among them developed infections, but the infecting pathogens were different from those found in the after-thawing cultures. Among the 1820 recipients who received sterile allografts, 21 (1.15%) had infections. The difference between the infection rate in recipients of sterile allografts (1.15%) and that in recipients of contaminated allografts (10%) was statistically significant (*P* < 0.01).

**Table 2:** Summary of recipients of bone allografts positive for bacterial culture after thawing.

Recipient	Age (years) / Sex	Procedure	Microorganisms	Infection	Antimicrobial therapy
1	58 / Male	PDPIPF	CoNS	No	No
2	55 / Male	PDPIPF	*Propionibacterium acnes*	No	No
3	78 / Male	PDPIPF	CoNS	No	No
4	52 / Female	ADAF	*Staphylococcus aureus*	No	No
5	73 / Female	PDPIPF	*Escherichia coli*	No	No
6	54 / Male	PDPIPF	CoNS	No	No
7	73 / Female	PDPIPF	*Bacillus* sp.	No	No
8	63 / Female	PDPIPF	*Candida albicans*	No	No
9	82 / Female	Revision THR	*Enterococcus* sp.	Yes^a^	Yes
10	68 / Female	PDPIPF	MRSA	Yes^b^	Yes
11	76 / Female	Revision ORIF	*Bacillus* sp.	No	No
12	71 / Male	Revision THR	CoNS	No	No
13	87 / Male	PDPIPF	*Bacillus* sp.	No	No
14	14 / Female	PIPF	CoNS	No	No
15	79 / Female	Revision THR	CoNS	No	Yes^c^
16	49 / Male	PDPIPF	CoNS	No	No
17	46 / Male	Revision THR	Gram-positive rods	No	No
18	70 / Female	PDPIPF	Gram-positive rods	No	Yes^c^
19	72 / Male	Revision ORIF	Non-fermentative GNB	No	No
20	55 / Female	ORIF for tibia plateau fracture	CoNS	No	No

^a^Blood: *Enterobacter cloacae*; Wound: *Escherichia coli*

^b^Wound: CoNS

^c^Positive results after-thawing cultures were noted early before discharging the patient, and intravenous antibiotics were administered for 7 days, followed by oral antibiotics for 14 days.

PDPIPF: posterior decompression, posterior instrumentation, and posterior fusion; THR: total hip replacement; ADAF: anterior decompression and anterior fusion; ORIF: open reduction and internal fixation; CoNS: coagulase-negative staphylococci; GNB: gram-negative bacilli

## Discussion

Over a period of 20 years (1991–2011), postoperative bacterial cultures revealed contamination in a total of 42 implanted allografts. Only six patients who received contaminated allografts developed infections later. With a single exception, the final pathogen identified in the wound culture of the allograft recipients differed from the pathogen identified in the after-thawing culture. In only one case was the wound culture (positive for *Candida* sp.) relatively compatible with the culture obtained after thawing (yeast-like organism) [9]. Many reports observed similar results and suggested that the allograft itself may not transmit infection from the donor to the recipient [2,3,6,9]. This is especially true in allografts obtained by standard arthroplasty from living donors selected by strict, established screening methods, which is the case for the allografts in our bone bank; the efficacy of prophylactic antibiotics may have also played a role in this sense. In most cases, contamination may have been introduced via specimen manipulation during the culture procedure and specimen processing in the microbiology laboratory. However, massive grafts with positive after-thawing culture might cause severe deep infection with the same bacteria [4]. Vehmeyer *et al*. suggest that when using massive grafts from cadaveric donors, blood culture results should be taken into account [10], while Angermann *et al*. suggest sterilization for all cadaveric bone grafts [11]. With the exception of the patient whose wound culture was positive for *Candida* sp., our results showed that, when using allografts from living donors and applying the allograft processing approach described here, a positive after-thawing culture does not correlate with the development of infection, and prophylactic antibiotic treatment is sufficient for preventing further infection. Based on our results, we may even speculate that antibiotic treatment may not be required for those patients receiving allografts with positive after-thawing culture. However, further prospective randomized controlled trials are warranted to confirm this speculation.

Hepatitis infection still represents the leading cause of discard in our hospital (73.2%), which is different from that in bone banks from Western countries [2–4,7]. The total discard rate decreased significantly in the second decade (from 23% to 18.4%), which may be due to a decrease in the number of HBsAg-positive donors. In July 1984, Taiwan’s mass hepatitis B vaccination program was launched, and in 1990, it was extended to include adults in addition to neonates born to HBsAg-positive mothers [12–14]. This vaccination program has been shown to play a significant role in decreasing the incidence of hepatitis B virus infection [13] and hepatocellular carcinoma in children [14]. The seroprevalence of the hepatitis B virus (HBV) in Taiwan between 1996 and 2005 was estimated at 17.3% [15]. According to our study, the seroprevalence was 12.7% (212/1674) in the first decade and 7.6% (245/3203) in the second decade, likely reflecting the changes in the national health policy toward HBV prevention and awareness of HBV carrier status among the Taiwanese population. As a result, many HBV carriers were excluded in the stage of recording donor history.

The rate of infection in allograft-related surgery was reported at 6.9%–12.2% [2,3,9]. Comparably, the rate of infection for allografts from our bank was relatively low (approximately 1.3%) in both the first [6] and the second decade. There are several explanations for this lower rate of infection. First, the infected patients were identified by our Center of Infection Control by connecting our bone bank database with records of in-hospital care. Only patients who needed intravenous antibiotics or surgical debridement were included in the estimation of infection rate, while those with superficial surgical site infections that could be treated successfully by oral antibiotics at outpatient clinics were not. Second, all our allografts were harvested during sterile surgery. Indeed, at our hospital, the infection rates for total knee replacement and total hip replacement were as low as 0.47% and 0.45%, respectively, in the second decade. Thus, we expect that our allografts were sterile during harvesting, and the chance of contamination would be lower. On the other hand, previous reports typically do not differentiate between allograft-related infections from living donors and those from cadaveric donors. It has been proven that allografts from cadaveric donors, especially massive grafts, are more likely to be contaminated and cause infection [3,4]. Therefore, previously reported infection rates may be skewed by the inclusion of a considerable number of higher risk allografts from cadaveric donors. Third, a substantial proportion of our allografts were used in scoliosis surgery, where the recipients were young and immunocompetent, and thus had better prognosis. Including such patients in a significant proportion may have contributed to the low infection rate noted in our study. Fourth, all allografts were washed with copious amounts of sterile normal saline and soaked in gentamicin solution (80 mg gentamicin in 200–300 mL normal saline, with the concentration between 0.27~0.4 mg/ml) for more than 10 min before implantation. Although Deijkers *et al*. [3] advocated that rinsing the graft with an antibiotic solution was not an effective decontamination method, they did mention that rinsing could reduce contamination by low-pathogenicity organisms and result in a reduced number of mildly contaminated allografts. Moreover, because our allografts come from living donors and are harvested under strict sterile arthroplasty procedures, there is the premise that antibiotic rinsing may indeed be efficient against low-pathogenicity organisms such as CoNS, especially in allografts with low microbial load. High-pathogenicity organisms typically originate from endogenous sources in the donor, usually from the upper respiratory or gastrointestinal tracts [16]; such individuals were identified during the stage of recording donor history or physical examination, and thus did not serve as donors. Fifth, almost all reports from other bone banks used first- or second-generation cephalosporins for rinsing the allografts; the antibacterial spectrum of cephalosporins is relatively similar to that of the prophylactic antibiotics used in the respective centers. In our bone bank, we used gentamicin, which has a different antibacterial spectrum, and may be more efficient against the low-pathogenicity organisms typically encountered in clinical practice in Taiwan. Moreover, in combination with prophylactic intravenous first-generation cephalosporins used in our hospital, rinsing with gentamicin may result in a lower infection rate because of a synergistic effect documented in detail elsewhere [17].

There is no consensus regarding the management of implanted allografts with positive after-thawing cultures. In the second decade, two patients (Table 2) with positive after-thawing cultures were identified early before discharge, and intravenous antibiotics were prescribed for 7 days, followed by oral antibiotics for 14 days according to the sensitivity test and upon the recommendation of our infectious disease specialist. Neither of these two patients developed infection. The other 18 patients whose allografts had positive after-thawing culture were followed-up carefully in an outpatient clinic; none of these patients developed infection, even though the positive culture result showed high-virulence microorganisms such as methicillin-resistant *S*. *aureus*, non-fermenting Gram-negative bacilli, and *Escherichia coli*. However, the post-operative infection rate was significantly higher in recipients of contaminated allografts than in recipients of sterile allografts (10% vs. 1.15%, *P* < 0.01). We thus conclude that the result of allograft after-thawing cultures is a good predictor for post-operative infection, but a poor predictor for the pathogen causing infection.

As in previous reports, we found that CoNS were the most common cultured bacteria [4,8,16]. CoNS were also the most common pathogens in the after-thawing culture, although they rarely contributed to infection in our patients. CoNS were historically thought to be skin commensal bacteria with low pathogenicity; thus, it is likely that CoNS accounted for a significant proportion of external contamination at the time of harvesting [16]. A growing number of recent studies consider CoNS to be one of the major nosocomial pathogens because of patient- and procedure-related aspects, such as infections in preterm newborns and foreign body-related infections [18], and the most common organism to cause nosocomial bloodstream infections [19]. Because of a lack of aggressive virulence properties, CoNS rarely attack a healthy host. However, patients who need allografts during revision surgeries are usually older and less immunocompetent, and are susceptible to infection when receiving CoNS-positive allografts. CoNS infection is challenging to treat because a significant number of strains are methicillin resistant; eventually, removal of any infected implanted device is required [18]. Our results reflect the potential hazard of CoNS infection in musculoskeletal allografting.

Our current study has two limitations. First, as suggested in many studies, we were unable to repeat the screening tests 3–6 months after allograft donation owing to poor compliance and under budgeting [3,4,11,20]. After screening the patient’s medical and social history and administering an HIV antibody test, the risk of HIV transmission is estimated at 0.009% [21], and freezing the bone graft further reduces this risk [22]. While HIV transmission can be difficult to manage, the prevalence of HIV in Taiwan is relatively low (0.019% in the general population) [23], and thus HIV is not expected to pose a significant risk. However, hepatitis B and C infections are more prevalent and take more time to make the serology detectable than HIV. Further study is needed to estimate the transmission rate of hepatitis B and C under the current operation protocols in our bone bank. The other limitation is that only one swab culture was used to detect contamination or infection of the bone allograft. The sensitivity and negative predictive value of single-swab cultures were reported at 10% and 9%, respectively [24]. However, Veen *et al*. reported that more heavily contaminated specimens will have fewer false-negative results, and swab culture techniques help identify such grafts [24]. Thus, we believe that, in the context of our harvesting, storage, and implantation protocols, a single swab culture may have been sufficient to detect heavily contaminated allografts likely to cause infection.

Our bone bank has worked well over the past 20 years. To the best of our knowledge, our study reports on the largest single-hospital living-donor series. The discard rate decreased significantly in the second decade, reflecting the success of Taiwan’s nationwide hepatitis B vaccination program. With a single exception, the positive after-thawing culture did not correlate with post-operative infection, even if the microorganism was highly pathogenic. Strict and established allograft screening and processing procedures, along with adequate prophylactic antibiotic treatment, are crucial for preventing infection.

## Supporting information

S1 Appendix Contraindications for bone donation. (DOCX)

S1 File Patient list. (XLS)

## References

[1] Joyce MJ, Greenwald AS, Rigney R Jr., Kennedy J, Toohey D, Heim CS, Rosier RN, Wong D. Musculoskeletal allograft tissue safety. 71st Annual Meeting of the American Academy of Orthopaedic Surgeons. 2004. Available: http://www.aaos.org/research/committee/biologic/bi_se_2004.pdf.

[2] Ivory JP, Thomas IH. Audit of a bone bank. J Bone Joint Surg Br. 1993;75: 355–357. 8496199 10.1302/0301-620X.75B3.8496199

[3] Sutherland AG, Raafat A, Yates P, Hutchison JD. Infection associated with the use of allograft bone from the north east Scotland Bone Bank. J Hosp Infect. 1997;35: 215–222. 9093920 10.1016/s0195-6701(97)90209-7

[4] Aho AJ, Hirn M, Aro HT, Heikkila JT, Meurman O. Bone bank service in Finland. Experience of bacteriologic, serologic and clinical results of the Turku Bone Bank 1972–1995. Acta Orthop Scand. 1998;69: 559–565. 9930097 10.3109/17453679808999255

[5] Liu JW, Chao LH, Su LH, Wang JW, Wang CJ. Experience with a bone bank operation and allograft bone infection in recipients at a medical centre in southern Taiwan. J Hosp Infect. 2002;50: 293–297. doi: 10.1053/jhin.2002.1192 12014903 10.1053/jhin.2002.1192

[6] Hou CH, Yang RS, Hou SM. Hospital-based allogenic bone bank—10-year experience. J Hosp Infect. 2005;59: 41–45. doi: 10.1016/j.jhin.2004.03.017 15571852 10.1016/j.jhin.2004.03.017

[7] Judas F, Teixeira L, Proenca A. Coimbra University Hospitals' bone and tissue bank: twenty-two years of experience. Transplant Proc. 2005;37: 2799–2801. doi: 10.1016/j.transproceed.2005.05.004 16182813 10.1016/j.transproceed.2005.05.004

[8] Chuang YC, Chen YC, Chang SC, Sun CC, Chang YY, Chen ML, et al Secular trends of healthcare-associated infections at a teaching hospital in Taiwan, 1981–2007. J Hosp Infect. 2010;76: 143–149. doi: 10.1016/j.jhin.2010.05.001 20663585 10.1016/j.jhin.2010.05.001 PMC7114588

[9] Tomford WW, Starkweather RJ, Goldman MH. A study of the clinical incidence of infection in the use of banked allograft bone. J Bone Joint Surg Am. 1981;63: 244–248. 7007391

[10] Vehmeyer SB, Bloem RM, Petit PL. Microbiological screening of post-mortem bone donors—two case reports. J Hosp Infect. 2001;47: 193–197. doi: 10.1053/jhin.2000.0836 11247679 10.1053/jhin.2000.0836

[11] Angermann P, Jepsen OB. Procurement, banking and decontamination of bone and collagenous tissue allografts: guidelines for infection control. J Hosp Infect. 1991;17: 159–169. 1675644 10.1016/0195-6701(91)90227-y

[12] Chen DS, Hsu NH, Sung JL, Hsu TC, Hsu ST, Kuo YT, et al A mass vaccination program in Taiwan against hepatitis B virus infection in infants of hepatitis B surface antigen-carrier mothers. JAMA. 1987;257: 2597–2603. 3573257

[13] Hsu HM, Chen DS, Chuang CH, Lu JC, Jwo DM, Lee CC, et al Efficacy of a mass hepatitis B vaccination program in Taiwan. Studies on 3464 infants of hepatitis B surface antigen-carrier mothers. JAMA. 1988;260: 2231–2235. 2971827

[14] Chang MH, Chen CJ, Lai MS, Hsu HM, Wu TC, Kong MS, et al Universal hepatitis B vaccination in Taiwan and the incidence of hepatocellular carcinoma in children. Taiwan Childhood Hepatoma Study Group. N Engl J Med. 1997;336: 1855–1859. doi: 10.1056/NEJM199706263362602 9197213 10.1056/NEJM199706263362602

[15] Chen CH, Yang PM, Huang GT, Lee HS, Sung JL, Sheu JC. Estimation of seroprevalence of hepatitis B virus and hepatitis C virus in Taiwan from a large-scale survey of free hepatitis screening participants. J Formos Med Assoc. 2007;106: 148–155. doi: 10.1016/S0929-6646(09)60231-X 17339159 10.1016/S0929-6646(09)60231-X

[16] Deijkers RL, Bloem RM, Petit PL, Brand R, Vehmeyer SB, Veen MR. Contamination of bone allografts: analysis of incidence and predisposing factors. J Bone Joint Surg Br. 1997;79: 161–166. 9020466 10.1302/0301-620x.79b1.7137

[17] Lawing CR, Lin FC, Dahners LE. Local injection of aminoglycosides for prophylaxis against infection in open fractures. J Bone Joint Surg Am. 2015;97: 1844–1851. doi: 10.2106/JBJS.O.00072 26582614 10.2106/JBJS.O.00072 PMC4642229

[18] Becker K, Heilmann C, Peters G. Coagulase-negative staphylococci. Clin Microbiol Rev. 2014;27: 870–926. doi: 10.1128/CMR.00109-13 25278577 10.1128/CMR.00109-13 PMC4187637

[19] Wisplinghoff H, Bischoff T, Tallent SM, Seifert H, Wenzel RP, Edmond MB. Nosocomial bloodstream infections in US hospitals: analysis of 24,179 cases from a prospective nationwide surveillance study. Clin Infect Dis. 2004;39: 309–317. doi: 10.1086/421946 15306996 10.1086/421946

[20] Yap PL. Viral transmission by blood, organs and tissues. J Hosp Infect. 1999;43: S137–144. 10658769 10.1016/s0195-6701(99)90076-2

[21] Buck BE, Malinin TI, Brown MD. Bone transplantation and human immunodeficiency virus. An estimate of risk of acquired immunodeficiency syndrome (AIDS). Clin Orthop Relat Res. 1989;(240): 129–316. 2645073

[22] Buck BE, Resnick L, Shah SM, Malinin TI. Human immunodeficiency virus cultured from bone. Implications for transplantation. Clin Orthop Relat Res. 1990;(251): 249–253. 2295182

[23] Fang CT, Hsu HM, Twu SJ, Chen MY, Chang YY, Hwang JS, et al Decreased HIV transmission after a policy of providing free access to highly active antiretroviral therapy in Taiwan. J Infect Dis. 2004;190: 879–885. doi: 10.1086/422601 15295691 10.1086/422601

[24] Veen MR, Bloem RM, Petit PL. Sensitivity and negative predictive value of swab cultures in musculoskeletal allograft procurement. Clin Orthop Relat Res. 1994;(300): 259–263. 8131346

