# Supplementary material for: Quality control processes in allografting: A twenty-year retrospective review of a hospital-based bone bank in Taiwan
Source: PLoS One. 2017 Oct 19;12(10):e0184809. doi: 10.1371/journal.pone.0184809 (PMC5648119; doi:10.1371/journal.pone.0184809)
Supplement: S1 Appendix — (DOCX) [file pone.0184809.s001.docx]

**Supporting Information**

**Appendix 1.** Contraindications for bone donation

1. History of active hepatitis or unexplained jaundice

2. Recent active systemic infection

3. Autoimmune disease

4. Malignant disease

5. Steroid therapy

6. Diabetes mellitus with non-healing chronic wound

7. Abnormal sexual history

8. History of drug abuse

9. Any sign/symptom of infection or fever of unknown origin
